# PReS-FINAL-1010: circulating micrornas in traps

**DOI:** 10.1186/1546-0096-11-S2-P8

**Published:** 2013-12-05

**Authors:** OM Lucherini, M Ferracin, V Fulci, M McDermott, G Merlini, I Muscari, F Magnotti, LJ Dickie, M Galeazzi, M Negrini, CT Baldari, L Obici, R Cimaz, L Cantarini

**Affiliations:** 1 Department of Medical Sciences, Surgical and Neuroscience. Rheumatology Unit., università di Siena, Siena, Italy; 2 Laboratory for Technologies of Advanced Therapies (LTTA) and Department of Morphology, Surgery and Experimental Medicine, University of Ferrara, Ferrara, Italy; 3 Dipartimento di Biotecnologie Cellulari ed Ematologia, Sezione di Genetica Molecolare, Sapienza, Università di Roma, Roma, Italy; 4 Leeds Institute of Rheumatic and Musculoskeletal Medicine, Leeds, UK; 5 Amyloid Research and Treatment Center, Fondazione IRCCS Policlinico San Matteo, and Department of Molecular Medicine, University of Pavia, Pavia, Italy; 6 Department of Life Sciences, Università di Siena, Siena, Italy; 7 Department of Pediatrics, Rheumatology Unit, Anna Meyer Children's Hospital and University of Florence, Florence, Italy

## Introduction

To the best of our knowledge circulating miRNAs in TRAPS, as well as in other monogenic autoinflammatory disorders have never been investigated.

## Objectives

To evaluate circulating microRNAs (miRNAs) levels in patients with tumor necrosis factor-receptor associated periodic syndrome (TRAPS), in comparison to healthy controls, and to correlate their levels to parameters of disease activity and/or disease severity.

## Methods

Expression levels of circulating miRNAs were measured by Agilent microarrays in 29 serum samples from 15 TRAPS patients carrying mutations known to be associated with high disease penetrance and 8 healthy controls. Differentially expressed and clinically relevant miRNAs were detected using GeneSpring GX software.

## Results

We identified a 6 miRNAs signature able to discriminate TRAPS from healthy controls. Moreover, 4 miRNAs were differentially expressed between patients treated with the interleukin (IL)-1 receptor antagonist anakinra and untreated patients. Of these, miR-92a-3p expression was found to be reduced in untreated patients, while its expression levels were similar to healthy controls in samples obtained during anakinra treatment. MiR-92b levels were inversely correlated with the number of fever attacks/year during the 1^st ^year from the index attack of TRAPS, while miR-377-5p levels were positively correlated with serum amyloid A (SAA) circulating levels.

## Conclusion

Serum miRNAs levels show a baseline pattern in TRAPS, and may serve as potential markers of response to therapeutic intervention.

## Disclosure of interest

None declared.

